# Complete mitochondrial genomes of endemic charr of the genus *Salvelinus* from Lake Nachikinskoe (Kamchatka, Russia)

**DOI:** 10.1080/23802359.2021.2010615

**Published:** 2021-12-28

**Authors:** Alla G. Oleinik, Andrey D. Kukhlevsky, Lubov A. Skurikhina

**Affiliations:** A.V. Zhirmunsky National Scientific Center of Marine Biology, Far Eastern Branch, Russian Academy of Sciences, Vladivostok, Russia

**Keywords:** Mitochondrial genome, mtDNA, charr genus *Salvelinus*, Taranetz charr, *Salvelinus taranetzi*

## Abstract

The complete mitochondrial genomes were sequenced from three individuals of the endemic charr *Salvelinus* sp. 4 [ from Lake Nachikinskoe (Kamchatka, Russia). The complete mitochondrial genomes were 16,654 bp in size; the genome organization and GC content (45.6%) was consistent with charr mitochondrial genomes published previously. The low level of sequence divergence detected between the *Salvelinus* sp. 4 and the GenBank *Salvelinus taranetzi* genomes indicated recent divergence and their origin from a common ancestor. Our results could play an essential role in resolving the conflict over current taxonomic status of endemic charr in genus *Salvelinus*.

Phylogenetic studies benefit significantly from decoding the complete mitochondrial genomes (mitogenomes) of narrow-ranged/endemic and disputed species of charrs, since the original descriptions of most of these are exclusively based on morphological features. Among such charrs is the endemic charr *Salvelinus* sp. 4 sensu Bogutskaya and Naseka (2004) represented by a single population in Lake Nachikinskoe (Kamchatka, Russia). Previous studies of *Salvelinus* sp. 4 are restricted to analyzing few mitochondrial genes (Oleinik, Skurikhina, Kukhlevsky, Bondar, 2019) and for further study and more precise phylogenetic analysis, it is most important to obtain the mitogenome of *Salvelinus* sp. 4.

We sequenced and described three mitogenomes of *Salvelinus* sp. 4 from Nachikinskoe Lake (53°01′ N, 157°50′ E). The specimens are stored in the collection of the Genetics Laboratory, NSCMB FEB RAS, Vladivostok, Russia (www.imb.dvo.ru) with accession numbers SPNA04.003, SPNA04.010, and SPNA04.019. Totally 23 pairs of primers were used (Table S1), which were designed with the mitoPrimer_V1 program (Yang et al. 2011) based on the charr mitogenomes that are public in GenBank. The sequenced fragments were assembled into mitogenomes and annotated by comparing with published charr mitogenome sequences using Geneious R11 (http://www.geneious.com/).

The size of the three mitogenomes of *Salvelinus* sp. 4 (MW181766, MW181767, and MW181768) was 16,654 bp. The genome organization was identical to that of typical salmon genomes, including 2 rRNA genes, 13 protein-coding genes, 22 tRNA genes, a light-strand replication origin (OL), and a control region (CR). The overall base composition was 28.0% A, 26.4% T, 17.0% G, and 28.6% C, and the GC content was 45.6%. This is consistent with previous results for charr mitogenomes (Balakirev et al. 2016; Oleinik, Skurikhina, Kukhlevsky, Semenchenko 2019). We detected six single-nucleotide and no length differences between the sequences MW181766, MW181768, and MW181767; total sequence divergence (*D*_xy_) was 0.00036 ± 0.00015. At the same time, 44 single-nucleotide substitutions were found between *Salvelinus* sp. 4 mitogenomes studied here and genomes of the congeneric species *S. taranetzi* (MK695630, and MK695631). Of these, 36 substitutions were found in overall protein coding sequences; five substitutions, in control region; two substitutions, in 12S rRNA; and one substitution in tRNA-Cys for MW181767 sequences. The degree of variability differed among protein coding genes, but variability of the NADH dehydrogenase subunit genes was highest for the compared mitogemones (52.3% of all variable sites). The lengths of tRNA coding genes ranged from 68 to 75 nucleotides, and total length of tRNA coding genes was equal (1560 bp) for the new mitogenomes and *S. taranetzi.*

The comparison of mitogenomes now obtained with 25 mitogenomes of related groups available in GenBank including genera *Salvelinus*, *Parahucho*, and *Salmo* point to a close relationship of *Salvelinus* sp. 4 to other charr species (Figure 1). All charr taxa showed similar phylogenetic relationships to those found by Oleinik et al. (2015). *Salvelinus* sp. 4 was phylogenetically positioned with charrs, and the level of divergence (*D*_xy_) between them and taxa within the genus was in the range from 0.00155 ± 0.00074 to 0.05236 ± 0.00564. The low sequence divergence was detected between the mitogenomes of *S. taranetzi* (MK695630, and MK695631) and our specimens (0.00242 ± 0.00061 on average). The level of sequence divergence among *Salvelinus* sp. 4 and *S. taranetzi* could be explained by their recent divergence and/or origin from a common ancestor.

**Figure 1.**
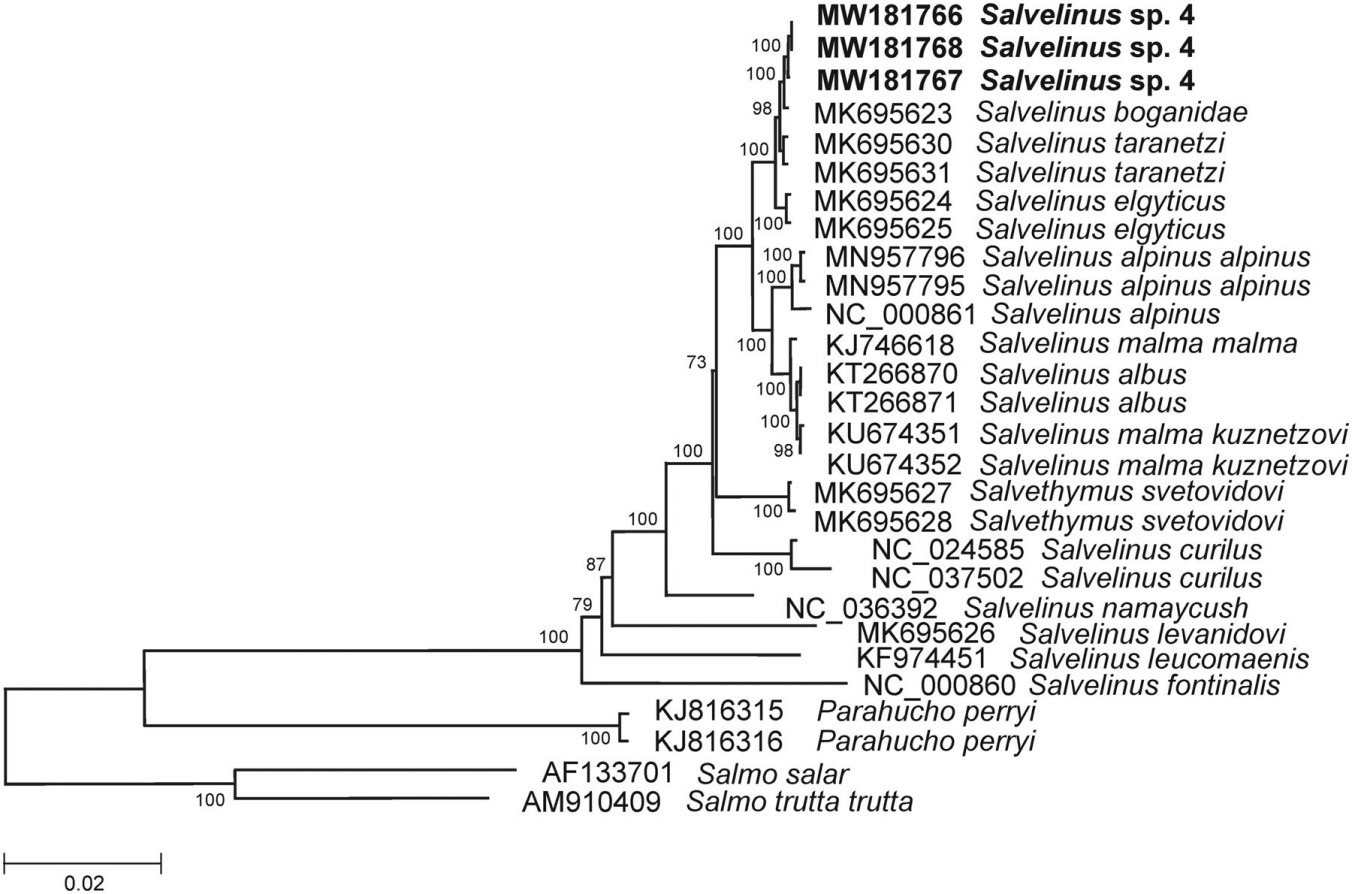
Maximum likelihood (ML) tree constructed on the comparison of complete mitochondrial genome sequences of *Salvelinus* sp. 4 from Lake Nachikinskoe (Kamchatka) and other GenBank representatives of the family Salmonidae. The tree is based on the GTR plus gamma plus invariant sites (GTR + G + I) model of nucleotide substitution. Genbank accession numbers for all sequences are listed in the figure. Numbers at the nodes indicate bootstrap probabilities from 1000 replications. Phylogenetic analysis was conducted in MEGA X (Kumar et al. 2018).

## Data Availability

The data that support the findings of this study are openly available in the National Center for Biotechnology Information database (NCBI/GenBank) at https://https.ncbi.nlm.nih.gov/, reference numbers MW181766, MW181767, and MW181768.
